# Piezoelectric‐Metal Phononic Crystal Enabling GHz Tunable Ultrahigh Q Quasi‐BIC Mode

**DOI:** 10.1002/advs.202513664

**Published:** 2025-10-17

**Authors:** Xuankai Xu, Jiawei Li, Ruoyu Wang, Ruihong Xiong, Yiwei Wang, Xiaoqin Shen, Tao Wu

**Affiliations:** ^1^ School of Information Science and Technology ShanghaiTech University Shanghai 201210 China; ^2^ School of Physical Science and Technology ShanghaiTech University Shanghai 201210 China; ^3^ State Key Laboratory of Quantum Functional Materials ShanghaiTech University Shanghai 201210 China; ^4^ Shanghai Engineering Research Center of Energy Efficient and Custom AI IC Shanghai 201210 China

**Keywords:** LiNbO_3_, phononic crystal, quasi‐BIC mode, tunable resonator

## Abstract

The integration of GHz‐frequency, high‐quality factor (*Q*), and electrically tunable acoustic resonators holds significant potential for advancing applications in quantum information technologies, microwave photonics, and reconfigurable RF systems. However, simultaneously achieving these three characteristics within a single, scalable platform remains a fundamental challenge. Here, the experimental demonstration of a GHz quasi‐BIC resonator in a piezoelectric thin‐film shear horizontal (SH) wave system, achieved through a structurally simple piezoelectric–metal phononic crystal (PnC) architecture on a LiNbO_3_ thin film, is reported. This approach enables leaky Fabry–Perot coupling mode and localized trapping quasi‐BIC mode. Without the need for deep etching or intricate patterning, a high room‐temperature quality factor of ≈6.5 × 10^4^ at ≈1 GHz in ambient air is achieved, corresponding to an *f* × *Q* product of ≈6.4 × 10^13^ Hz at quasi‐BIC mode. Furthermore, efficient electrical tunability is demonstrated via low–voltage (0.6 V) electrothermal modulation of the PnC structure, enabling a reversible transition between trapped and transmission states and yielding a high‐contrast amplitude modulation of 47.75 dB. This work opens new directions for scalable on‐chip phononic circuits in quantum acoustics, reconfigurable RF systems, and signal processing applications.

## Graphical abstract

This study introduces a piezoelectric–metal phononic crystal design based on suspended LiNbO_3_ thin film that realizes quasi‐BIC resonance. The device achieves GHz operation with an ultrahigh quality factor (≈6.5 × 10^4^) and efficient electrical tunability at low voltage. Combining performance, scalability, and reconfigurability, it opens new possibilities for quantum acoustics, reconfigurable RF systems, and phononic signal processing.

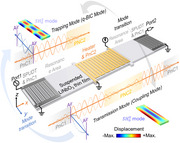

## 1 Introduction

Piezoelectric acoustic wave devices have a long‐standing history that dates to the pioneering work on surface acoustic waves (SAWs) in the 1960s by White and Voltmer, who first demonstrated interdigital transducers (IDTs) on piezoelectric substrates.^[^ ^1^ ^]^ Since then, SAW,^[^ ^2^, ^3^, ^4^ ^]^ bulk acoustic wave (BAW)^[^ ^5^, ^6^, ^7^, ^8^ ^]^ and lamb wave^[^ ^9^, ^10^, ^11^ ^]^ devices have been extensively studied and widely deployed in areas ranging from RF filters and oscillators to sensing and signal processing, as documented in numerous foundational textbooks and reviews.^[^ ^12^, ^13^, ^14^, ^15^ ^]^ The strong electromechanical coupling, compatibility with planar fabrication, and frequency scalability of piezoelectric acoustic devices have established them as indispensable components in classical electronics and communications. Over the decades, significant progress has been made in extending acoustic device concepts from low‐MHz to multi‐GHz operation, enabling compact and high‐performance RF front‐ends for wireless communications.

Beyond their well‐established roles, piezoelectric acoustic devices are emerging as key enablers for next‐generation technologies such as quantum information processing,^[^ ^16^, ^17^, ^18^ ^]^ microwave photonics,^[^ ^19^, ^20^, ^21^ ^]^ and advanced RF systems.^[^ ^22^, ^23^, ^24^ ^]^ For these applications, acoustic resonators that simultaneously support GHz operation, ultrahigh *quality factor (Q)*, and electrical tunability are especially desirable: GHz frequencies are vital for superconducting qubits,^[^ ^16^, ^17^ ^]^ RF communications,^[^ ^25^, ^26^, ^27^ ^]^ and high‐speed acousto‐optic modulation^[^ ^19^, ^20^, ^21^ ^]^; high‐*Q* factors enhance spectral selectivity^[^ ^28^, ^29^ ^]^ and signal‐to‐noise ratio;^[^ ^30^, ^31^ ^]^ and electrical tunability provides dynamic reconfigurability in filters,^[^ ^32^, ^33^ ^]^ modulators,^[^ ^22^, ^23^, ^34^ ^]^ and switches.^[^ ^35^, ^36^, ^37^ ^]^ Achieving all three attributes in a single platform could unlock transformative capabilities—from scalable quantum transduction to adaptive RF front‐ends and chip‐scale phononic processors.^[^ ^24^, ^38^, ^39^ ^]^ Yet, such devices remain scarce, underscoring a critical challenge in advancing integrated phononic platforms for both classical and quantum technologies.

Phononic crystals (PnCs) provide a powerful route for engineering acoustic wave confinement and play a central role in realizing bound states in the continuum (BICs)^[^ ^40^, ^41^, ^42^, ^43^ ^]^ by tailoring band structures and symmetry properties. BIC can suppress radiative losses through symmetry protection or destructive interference, theoretically allowing infinite‐*Q* modes. While BIC concepts have been widely explored in optics^[^ ^44^, ^45^, ^46^, ^47^, ^48^ ^]^ and bulk acoustic systems,^[^ ^49^, ^50^, ^51^, ^52^, ^53^ ^]^ their implementation in piezoelectric thin‐film devices at microwave frequencies remains highly challenging. Existing strategies often rely on complex etched architectures, such as partially etched PnC slabs or fully etched holes and pillars which demand stringent fabrication precision.^[^ ^54^, ^55^, ^56^, ^57^, ^58^ ^]^ In practice, pattern misalignments and etching sidewall roughness induce scattering losses,^[^ ^56^, ^59^, ^60^ ^]^ limiting the achievable *Q* factors. Furthermore, such architectures are difficult to scale into the multi‐GHz regime and inherently lack real‐time electrical tunability, restricting their use in dynamic RF and quantum systems. These challenges underscore the need for a structurally simple, fabrication‐friendly, and electrically tunable BIC platform capable of combining GHz operation with ultrahigh *Q* in chip‐scale devices.

In this work, we report the first experimental realization of a GHz quasi‐BIC resonator in a piezoelectric thin‐film shear horizontal (SH) wave system, leveraging a simple piezoelectric‐metal PnC architecture. By patterning a metal‐PnC lattice atop a LiNbO_3_ thin film, we engineer phononic band structures that support both leaky Fabry–Perot coupling modes and localized trapping states. Through interference between the fundamental and higher‐order SH modes, we achieve quasi‐BIC formation by decoupling the acoustic energy from the radiation channel. Without resorting to partial etching or intricate structural tuning, we achieve a high room‐temperature quality factor of ≈6.5 × 10^4^ at ≈1 GHz in ambient air, yielding an *f* × *Q* product of 6.4 × 10^13 ^Hz — surpassing the best reported values in far more complex systems. More importantly, we demonstrate the tunability of quasi‐BIC state. By applying a low‐voltage (0.6 V) electrothermal bias to the piezoelectric‐metal PnC, we modulate the acoustic interference condition and reversibly transition the mode from trapped to transmission, which results in a high‐contrast amplitude modulation of 47.75 dB. Our work proves that ultrahigh‐*Q* and electrically tunable quasi‐BIC modes can be realized using a structurally simple, lithography‐friendly design, bypassing many of the limitations that have stalled prior efforts. This approach offers a practical, scalable platform for high‐*Q* filters, reconfigurable phononic networks, and quantum signal processors, bringing BIC‐enabled acoustics closer to real‐world integration.

## 2 Design and Phononic Confinement of the Metal–Piezoelectric PnC Resonator

**Figure 1:**
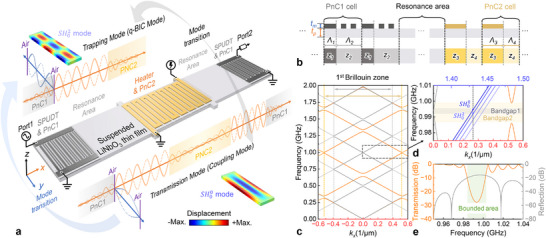
a) Schematic of the 1D metal–piezoelectric PnC resonator. SH waves are generated and detected by SPUDTs via the piezoelectric effect. The SPUDTs also serve as outer reflectors (PnC1), forming a phononic bandgap. The inner reflectors (PnC2) define the cavity boundary and simultaneously function as integrated heaters for thermoelastic modulation. b) Cross‐sectional view of the resonant cavity, showing the periodic arrangement of PnC1 and PnC2. c) Calculated band structures of PnC1 (grey) and PnC2 (orange), exhibiting overlapping bandgaps centered near 1 GHz. This spectral overlap enables confinement of acoustic waves within the cavity. d) Selective trapping of shear horizontal standing wave modes, including $SH_{0}^{0}$ (no nodal points along the y‐direction) and $SH_{0}^{2}$ (two nodal points along the y‐direction) mode, within the overlapping bandgap region. e) Simulated reflection spectrum of PnC1 (10 unit cells) and transmission spectrum of PnC2 (31 unit cells). The shaded region indicates the overlapping bandgap that defines the acoustic confinement window.

To realize GHz‐frequency acoustic resonances energy coupling channel, we designed a 1D metal–piezoelectric PnC resonator based on a suspended LiNbO_3_ thin film. As illustrated in **Figure** **1**a, SH waves are excited and detected by single‐phase unidirectional transducers (SPUDTs), which also serve as outer reflectors (PnC1), forming a phononic bandgap. The inner reflectors (PnC2) define the cavity boundaries and simultaneously act as electrothermal heaters, enabling dynamic tuning of the acoustic response (Figure 1b). The selection for piezoelectric and metal matrials is 290 nm Z‐cut LiNbO_3_ thin film and 100 nm aluminum, respectively, the detailed design parameters are provided in Note S1 (Supporting Information). Figure 1c shows the simulated band structures of PnC1 (grey) and PnC2 (orange), both exhibiting phononic bandgaps centered near 1 GHz. Their spectral overlap enables strong confinement of SH waves within the acoustic cavity. The cavity geometry was engineered to achieve the interference required for quasi‐BIC formation. The resonator length *L_R_* ​ was selected such that the fundamental lateral mode $SH_{0}^{0}$​ falls inside the overlapping bandgap, while the resonator width *W_R_* was adjusted so that the higher‐order lateral mode $SH_{2}^{0}$​ also resides in the same frequency window, the black dashed line marks the in‐plane wavevector *k_x_* selected for resonance (Figure 1d). This design ensures that the two confined modes possess close spectral proximity and spatial overlap, thereby creating favorable conditions for destructive interference.

When the $SH_{0}^{0}$ mode couples through PnC2, it supports a transmission channel that radiates into the continuum. In contrast, when the $SH_{0}^{0}$​ and $SH_{2}^{0}$​ modes hybridize, their destructive interference at the coupling interface of PnC2 suppresses leakage, trapping acoustic energy within the cavity which forms a confined quasi‐BIC. This mechanism resembles the Friedrich–Wintgen type of interference‐induced BICs,^[^ ^61^, ^62^ ^]^ where destructive mode interference suppresses leakage channels and leads to strong confinement of acoustic energy. Figure 1e confirms the bandgap overlap and the selective trapping behavior through simulated reflection and transmission spectra.

## 3 Engineering Quasi‐BIC Mode Through Phononic Coupling

**Figure 2:**
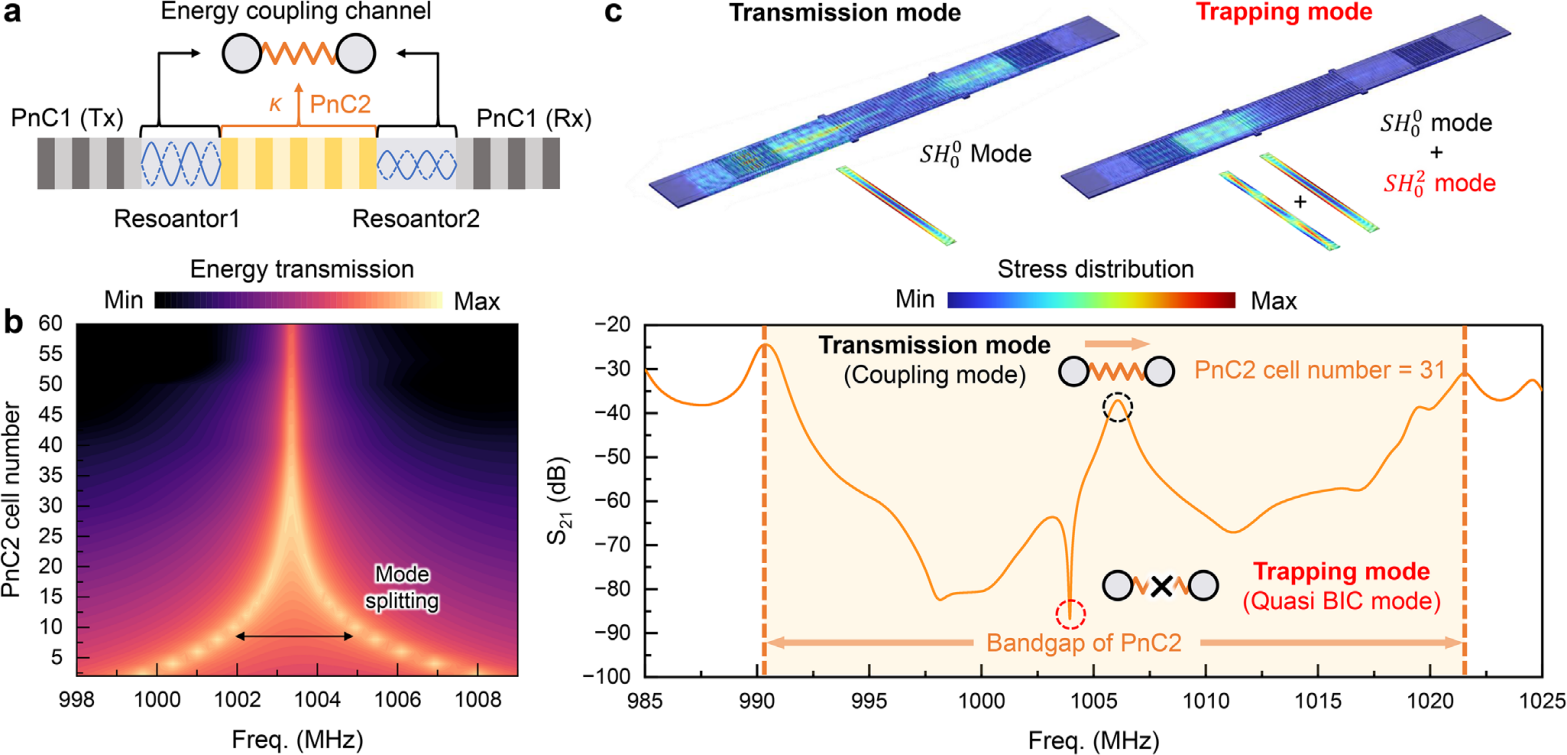
a) Two SH wave resonators (Resonator 1 and Resonator 2) are coupled through a central PnC2 lattice. PnC1 (left and right) defines the resonator boundaries by reflecting SH waves, while PnC2 serves as a tunable acoustic spring that mediates inter‐cavity coupling. b) A reduced number of unit cells weakens confinement and increases the inter‐resonator coupling strength (*κ*), resulting in mode splitting. c) Simulated frequency response within the PnC2 bandgap (shaded region) demonstrates a transmission channel that supports coupling of the $SH_{0}^{0}$ mode between the two resonators. Destructive interference between the $SH_{0}^{0}$ and $SH_{0}^{2}$ modes suppresses radiation loss, decoupling from the radiation channel, enabling the formation of a hybridized quasi‐BIC with an enhanced *Q*. Simulated stress profiles (top) illustrate the transmission mode with delocalized energy (left) and the quasi‐BIC mode with localized energy confinement (right).

To construct interference‐induced quasi‐BIC modes, we implemented a coupled‐resonator structure consisting of two SH wave cavities connected via a central PnC2 region (**Figure** **2**a). In this configuration, the outer PnC1 segments function as reflective boundaries, while the intermediate PnC2 serves as a phononic coupler. By tuning the number of PnC2 unit cells, the effective stiffness of the coupling region can be adjusted, allowing the construction of a coupled Fabry–Pérot resonator. The detailed design of this configuration is provided in Note S2 (Supporting Information). As the number of PnC2 unit cells is reduced (Figure 2b), the inter‐cavity coupling strength *κ* increases, enhancing modal interaction and leading to mode splitting. Finite‐element simulations (Figure 2c) show that, within the overlapping phononic bandgap, the fundamental $SH_{0}^{0}$ mode supports a transmission channel across the coupled cavities. However, when the $SH_{0}^{0}$ and higher‐order $SH_{2}^{0}$ modes become nearly degenerate, their destructive interference gives rise to a hybridized mode that decouples from the continuum—forming a quasi‐BIC with a substantially enhanced *Q* of the resonator. The simulated stress field distributions illustrate the distinction between a delocalized transmission mode and a spatially confined quasi‐BIC mode, confirming the role of phononic coupling and modal interference in achieving acoustic confinement.

## 4 Experimental Observation of High‐Q Acoustic Trapping

**Figure 3:**
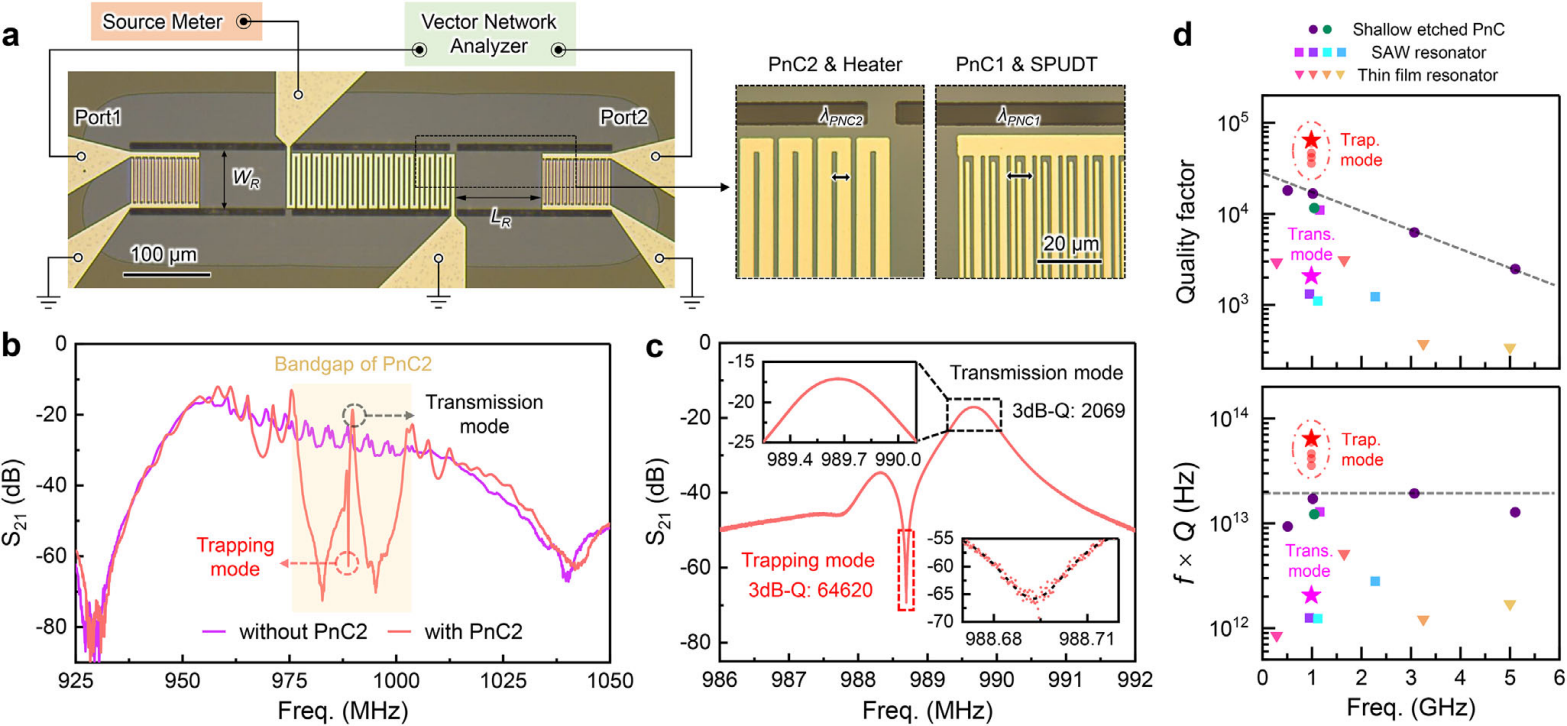
a) Optical micrograph of the fabricated resonator, with inset images highlighting the periodic structures of PnC1 and PnC2. Transmission measurements (S_21_) are performed using a vector network analyzer (VNA), while a source meter supplies heating power to enable thermal tuning of the PnC2 lattice. b) Measured S_21_ spectra with and without the PnC2 structure (0 V input from the source meter). c) Magnified view of the S_21_ spectra showing a substantial *Q*‐factor enhancement—from 2,069 (transmission mode) to 64,620 (quasi‐BIC mode). d) Comparison of the achieved Q‐factor (highlighted by a star) with state‐of‐the‐art LiNbO_3_‐based resonators reported in the literature, and benchmarking of the frequency–quality factor product (*f* × *Q*) for the same set of devices.

We experimentally validated the quasi‐BIC resonator design through room‐temperature transmission measurements on a fabricated device (**Figure** **3**a). The resonance region has a length 𝐿_𝑅_ = 101.5 µm and a width *W*_𝑅_ = 70 µm; the unit cell lengths of PnC1 and PnC2 are *λ_PnC1_* = 8 µm and *λ_PnC2_* = 6 µm, respectively. Transmission spectra (S_21_) were recorded using a vector network analyzer (VNA), while a source meter supplied DC heating power to the integrated PnC2 heaters. Further details on device fabrication and the experimental setup are provided in the Methods section. As shown in Figure 3b, the control device without PnC2 exhibits no bandgap, allowing free propagation of SH waves. In contrast, incorporation of PnC2 leads to the formation of a clear transmission bandgap (shaded region), within which two distinct resonances emerge: a broad transmission mode (coupling mode) and a narrow trapping mode (quasi‐BIC mode). Comparative measurements with and without PnC2 are presented in Note S4 (Supporting Information).

A magnified view of S_21_ (Figure 3c) reveals a dramatic quality factor enhancement—from 2069 for the transmission mode to 64620 for the quasi‐BIC mode—providing direct evidence of radiation suppression via engineered modal interference. Notably, the achieved high *Q* factor in ambient air primarily originates from the quasi‐BIC mechanism, which effectively confines the acoustic energy within the resonant region composed of bare LiNbO_3_ thin film (resonator region). Under this condition, the dominant loss mechanism is determined by the intrinsic material *Q* of LiNbO_3_.^[^ ^59^, ^60^ ^]^ Since the intrinsic *Q* of LiNbO_3_ is considerably higher than that of Al,^[^ ^59^ ^]^ any resonance mode with strong acoustic field overlap in the electrode region (e.g., the transmission‐like mode) suffers from substantial damping due to the low intrinsic *Q* of Al. In contrast, for the quasi‐BIC mode, where the energy is predominantly localized in the bare LiNbO_3_ region, the overall *Q* is governed by the intrinsic properties of LiNbO_3_, thus enabling the realization of the high *Q* values observed in our measurements. We evaluated reproducibility by measuring five nominally identical devices from the same batch. As shown in Figure 3d, for the quasi‐BIC trapping mode, the *Q*‐factor ranged from ≈3.6 × 10^4^ to ≈6.5 × 10^4^ (mean value: ≈5.0 × 10^4^), corresponding to *f* × *Q* products between 3.5 × 10^13^ and 6.4 × 10^13^ Hz. These values surpass previously reported benchmarks while maintaining a fabrication‐friendly architecture that avoids deep etching or subwavelength patterning. The performance of additional fabricated devices is detailed in Note S5 (Supporting Information), and a more comprehensive comparison with the state‐of‐the‐art devices is provided in Note S7 and Table S1 (Supporting Information).^[^ ^55^, ^57^, ^63^, ^64^, ^65^, ^66^, ^67^, ^68^, ^69^, ^70^ ^]^

## 5 Dynamic Tuning of the Transmission and Trapping Modes

**Figure 4:**
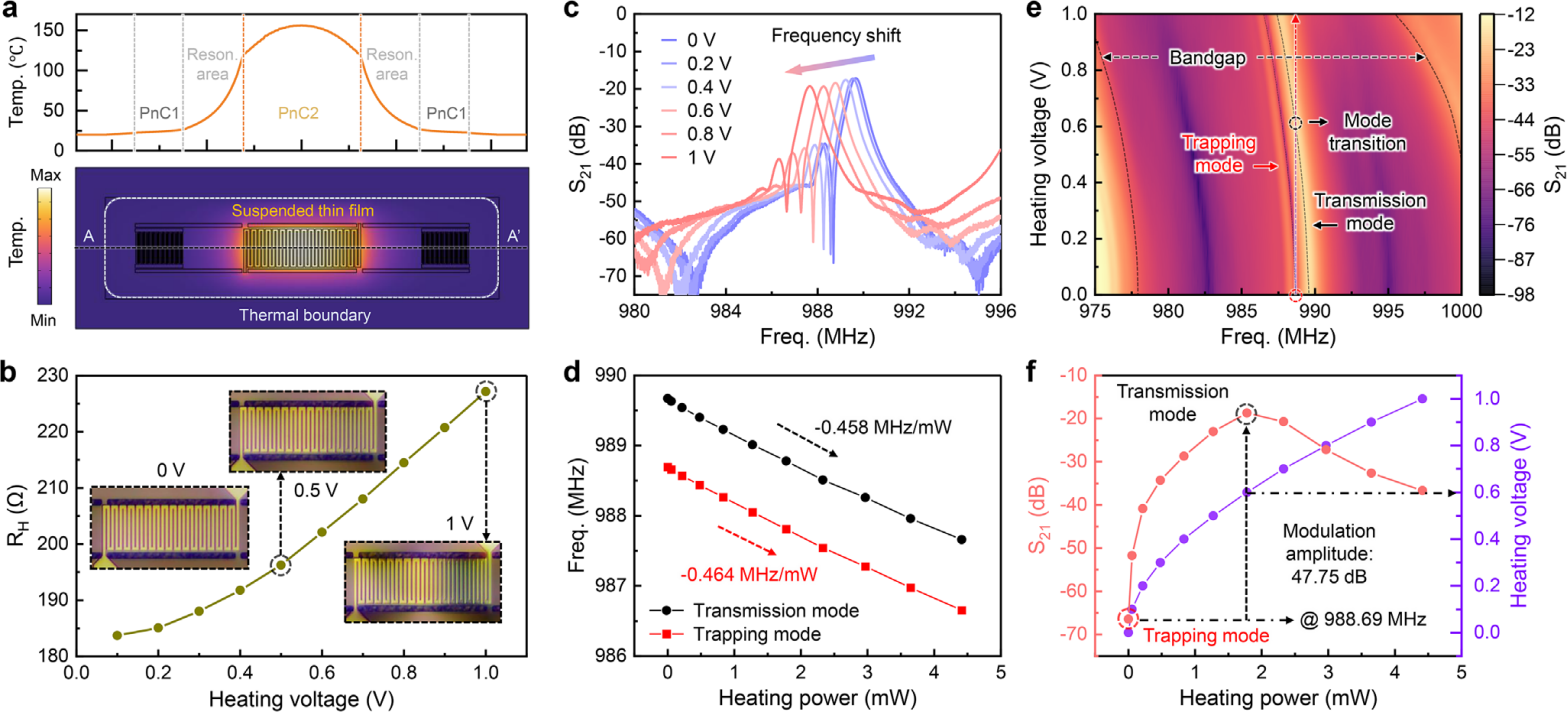
a) Simulated thermal profile of the resonator under Joule heating, illustrating the localized temperature distribution: PnC2 acts as the central heating zone, the resonance region is moderately heated, while PnC1 remains near ambient temperature. b) Measured resistance of the integrated heater as a function of input voltage. Optical micrographs show progressive thermal expansion of the PnC2 region with increasing voltage (0 to 1 V). c) Transmission mode, and quasi‐BIC mode redshift as the applied heating voltage increases. d) Extracted thermal sensitivity of the quasi‐BIC and transmission modes, showing frequency shifts of −0.464 and −0.458 MHz Mw^−1^, respectively. e) Modulation spectra under stepwise voltage application demonstrate redshifts of both the PnC2 bandgap and resonance modes. f) Mode transition under thermal tuning, the quasi‐BIC transitions into the transmission mode at 0.6 V, at 988.69 MHz.

To evaluate the active tunability of the resonator, we applied voltage‐controlled Joule heating to the PnC2 region and monitored the resulting thermal and spectral response. Simulated thermal profiles (**Figure** **4**a) reveal that heating is localized within PnC2, where a heating voltage of 1 V is applied, while the resonance cavity is moderately heated and the PnC1 reflectors remaining near ambient temperature. The resistance of the integrated heater increases with voltage (Figure 4b), while optical micrographs show progressive thermal expansion of PnC2 from 0 to 1 V. Thermal tuning results in consistent frequency shifts of both the transmission and trapping modes (Figure 4c), with extracted thermal sensitivities of −0.458 and −0.464 MHz mW^−1^, respectively (Figure 4d). As shown in Figure 4e, stepwise voltage application shifts both the phononic bandgap and the resonance frequencies, with the bandgap exhibiting the largest shift due to direct heating. Thermal modulation drives a transition from the trapping mode to the transmission mode at an input voltage of 0.6 V (Figure 4f), producing a pronounced change in transmission amplitude. This voltage corresponds to a power consumption of only 1.78 mW and, according to simulations, induces a 52.7 °C temperature rise in the PnC2 region, highlighting the high efficiency of the thin‐film electrothermal heating. At 988.69 MHz, the device achieves a modulation depth of 47.75 dB, demonstrating its potential for low‐power signal control in reconfigurable RF and phononic systems. In addition to static tuning, we also investigated AC thermal modulation, which showed millisecond‐scale rise and fall times, considerably faster than typical SAW‐based platforms.^[^ ^71^ ^]^ The detailed characterization and discussion are provided in Note S6 (Supporting Information).

## 6 Discussion

In this work, we demonstrate a structurally simple yet highly effective approach to achieving GHz‐frequency, ultrahigh‐*Q*, and electrically tunable acoustic resonators using a metal–piezoelectric PnC on a suspended LiNbO_3_ thin film. Through phononic bandgap engineering and modal interference between SH modes, we realized a quasi‐BIC resonance with high *Q*‐factors ranged from ≈3.6 × 10^4^ to ≈6.5 × 10^4^ (mean value: ≈5.0 × 10^4^) and the corresponding *f* × *Q* products between 3.5 × 10^13^ and 6.4 ×  10^13^ Hz, comparable to or better than other complex etched systems. Meanwhile, the PnC2 region also functions as an integrated electrothermal modulator, enabling low‐voltage, reversible tuning between trapped and transmission states. The observed 47.75 dB modulation depth and MHz Mw^−1^‐level thermal sensitivities confirm the resonator potential for active control in reconfigurable RF and phononic circuits.

Unlike prior BIC‐based devices that rely on precise nanofabrication or aggressive etching to define resonant modes, our design is lithography‐friendly and fabrication‐tolerant, making it scalable for large‐area integration. This advantage arises because the resonant cavity is primarily formed in the suspended non‐patterned LiNbO_3_ region, while the lithographically defined features (SPUDTs and PnCs) only serve as excitation and confinement elements. As a result, the lithography requirements are relatively relaxed, and the acoustic energy remains largely stored in the pristine LiNbO_3_ film, thereby preserving its intrinsically high *Q*‐factor. The proposed method can be extended to various acoustic platforms incorporating heterogeneous materials to realize high‐*Q* tunable acoustic cavities.^[^ ^72^, ^73^ ^]^ While the present work focuses on thermoelastic modulation as the tuning mechanism, the concept can be possibly extended to other approaches such as RF‐power loading,^[^ ^74^ ^]^ acoustoelectric modulation,^[^ ^22^ ^]^ and electro‐acoustic tuning.^[^ ^23^ ^]^ In future studies, we aim to further refine the theoretical framework of this system, including detailed investigations into the coupling mechanisms and avoided‐crossing behavior of FW‐BIC, to provide comprehensive support for realizing such high‐performance acoustic resonators across diverse material platforms and high‐frequency applications. The modularity of the architecture also supports future extensions to quantum acoustics, nonreciprocal devices, and programmable phononic networks. Overall, this work establishes a new platform for high‐performance and tunable acoustic devices, combining simplicity, performance, and control in a form readily adaptable to practical applications.

## 7 Experimental Section

### Device Fabrication

The fabrication process began with a 750 nm Z‐cut LiNbO_3_ film bonded on a silicon substrate. The film was thinned to a target thickness of 290 nm using ion beam etching (IBE, scia Mill 150) operated at a microwave ion source power of 260 W and a beam voltage of 400 V for 74 min. The thickness was measured with a film thickness mapper (Filmmetrics F50‐UV) at 15 uniformly distributed points across a 10 mm × 6 mm area, yielding a final value of 292.4 nm with a root‐mean‐square (RMS) variation of 4.6 nm. The effect of thickness variation on the *Q* factor is analyzed by FEA and presented in Note S3 (Supporting Information). The release boundaries of the suspended device were defined by patterning photoresist (AZ5214) using a maskless aligner (MLA 150), followed by the same IBE etching for 90 min to achieve complete penetration through the LiNbO_3_ film. The SPUDT electrodes and PnC mirrors were then defined in a second lithography step using the same photoresist and aligner. A 100 nm Al film was deposited by sputtering (PRO Line PVD 75) and patterned through a lift‐off process. Finally, the underlying silicon substrate was removed by XeF_2_ dry etching (SPTS XeF_2_ Etcher), producing a 60 µm‐deep air cavity beneath the suspended LiNbO_3_ membrane.

### Finite‐Element Modeling

The frequency response and stress distribution of transmission mode and coupling mode were simulated using finite‐element methods with COMSOL Multiphysics software, a widely used tool for simulating acoustic thin‐film resonators. The solid mechanics and electrostatic modules were coupled in the Piezoelectric Effect Multiphysics module, enabling computation of both mechanical and electrical responses. The software solves elastic wave equations in a linear piezoelectric medium with strain‐charge coupling. Material properties for the simulation were sourced from the COMSOL Material Library.

### Experimental Setup

The S‐parameters of the devices were measured using a vector network analyzer (Keysight N5234B) with an RF probe station (MPI TS150) equipped with two GSG RF probes (MPI T26A, 150 µm pitch). For DC thermal tuning, a DC bias voltage was applied through two DC probes connected to a precision source measure unit (Keysight B2901A). For AC thermal tuning, a waveform generator (Keysight 33622A) was used to provide the AC modulation square wave.

## Conflict of Interest

The authors declare no conflict of interest.

## Author Contributions

X.X., J.L., and R.W. contributed equally to this work and are co‐first authors. X.X. conceived the concept, carried out theoretical analysis, contributed to device fabrication and measurements, and wrote the manuscript; J.L. performed the finite element simulations and participated in device fabrication and measurements; R.W. conducted phononic band structure calculations, contributed to theoretical discussions, and assisted in manuscript preparation; R.X. and Y.W. assisted in device fabrication and measurements; X.S. and T.W. supervised the project, provided technical guidance, and revised the manuscript. All authors discussed the results and contributed to the final version of the manuscript.

## Supporting information



Supporting Information

## Data Availability

The data that support the findings of this study are available from the corresponding author upon reasonable request.
