# Supplementary material for: Piezoelectric‐Metal Phononic Crystal Enabling GHz Tunable Ultrahigh Q Quasi‐BIC Mode
Source: Adv Sci (Weinh). 2025 Oct 17;13(6):e13664. doi: 10.1002/advs.202513664 (PMC12866841; doi:10.1002/advs.202513664)
Supplement: Supplementary file 1 — Supporting Information [file ADVS-13-e13664-s001.docx]

Supplementary Information for “Piezoelectric-Metal Phononic Crystal enabling Tunable GHz Ultrahigh Q Quasi-BIC mode”

Xuankai Xu^1#^, Jiawei Li^1#^, Ruoyu Wang^2#^, Ruihong Xiong^1^, Yiwei Wang^1^, Xiaoqin Shen^2,3*^ and Tao Wu^1,4*^

^1^School of Information Science and Technology, ShanghaiTech University, Shanghai, 201210, China.

^2^School of Physical Science and Technology, ShanghaiTech University, Shanghai, 201210, China

^3^State Key Laboratory of Quantum Functional Materials, ShanghaiTech University, Shanghai 201210

^4^Shanghai Engineering Research Center of Energy Efficient and Custom AI IC, Shanghai, 201210, China

^#^These authors contributed equally to this work.

^*^E-mail: shenxq@shanghaitech.edu.cn; wutao@shanghaitech.edu.cn

**Supplementary Note 1: The Detailed structure and design of PnC1 and PnC2**

Figure S1 summarizes the structural parameters and simulated results. To achieve efficient acoustic confinement and selective mode coupling, the phononic band structures of PnC1 and PnC2 must be precisely engineered. Both structures are designed as one-dimensional phononic crystals consisting of alternating regions with different acoustic impedances. **Figure S1 (a)** shows the detailed design of PnC1 and PnC2, PnC1 has a unit cell size of *λ_PnC1_* = 8 μm, while PnC2 uses *λ_PnC2_* = 6 μm. The propagation velocity of the excited acoustic mode ($SH_{0}$) was obtained through finite element method simulations. **Figure S1 (b)–(c)** illustrates the dependence of $SH_{0}$ phase velocity on film thickness and metallization condition. For $SH_{0}$ waves under electrically open conditions, the phase velocity in 290 nm LiNbO₃ was determined to be approximately 4355 m/s. In regions with aluminum coverage, the velocity decreases dramatically due to electric short condition, increased mass loading and young’s modulus contrast.

To achieve acoustic wave confinement between phononic crystals (PNC1 and PNC2), their bandgap frequencies must align precisely. The phononic band structures discussed here are calculated using the transfer matrix method. The region labeled *Λ*_2_ in PNC1 is not composed of a single homogeneous material. Consequently, the acoustic velocity in the *Λ*_2_ region is an intermediate value determined by the area fraction of aluminum electrodes. This effective velocity is expressed as $v_{\Lambda2}=d*v_{LN}+\left( 1-d \right){*v}_{LN+AL}.$ where the parameter $d$ represents the aluminum electrode coverage ratio within the *Λ_2_* region.

$$\cos\left( kd \right)=\cos\left( \frac{\omega d_{1}}{v_{1}} \right)*\cos\left( \frac{\omega d_{2}}{v_{2}} \right)- \frac{1}{2}*\left( \left( \frac{Z_{1}}{Z_{2}} \right)+ \left( \frac{Z_{2}}{Z_{1}} \right) \right)*\sin\left( \frac{\omega d_{1}}{v_{1}} \right)*\sin\left( \frac{\omega d_{2}}{v_{2}} \right)$$

The dispersion relation of a one-dimensional phononic crystal is described by the equation mentioned above. In this equation, Z_1_ and Z_2_​ represent the acoustic impedances of the two materials, which depend on their densities ($\rho$) and acoustic velocities ($v$) according to the relation$Z=\rho v$.

The reflectivity and transmission characteristics of PnC1 for varying numbers of unit cells (N_PnC1_ = 10, 50, 100) and PnC2 for varying numbers of unit cells (N_PnC2_ = 30, 50, 100) are shown in **Figure S1 (d)–(e)**. As the number of layers increases, the reflectivity at the center frequency of the bandgap rises, while the bandwidth of the reflected frequency range decreases. For practical implementation, the number of unit cells was chosen as N_PnC1_​=10 for the outer reflectors and N_PnC2_​=31 for the inner reflectors. This configuration provides a broader reflection bandwidth at the outer boundaries and a narrower but stronger reflection within the inner cavity region, ensuring sufficient spectral overlap between the two phononic mirrors and enabling robust acoustic confinement.

**Supplementary Note 2: 2.5D FEA simulation of the metal-piezoelectric PnC resonator**


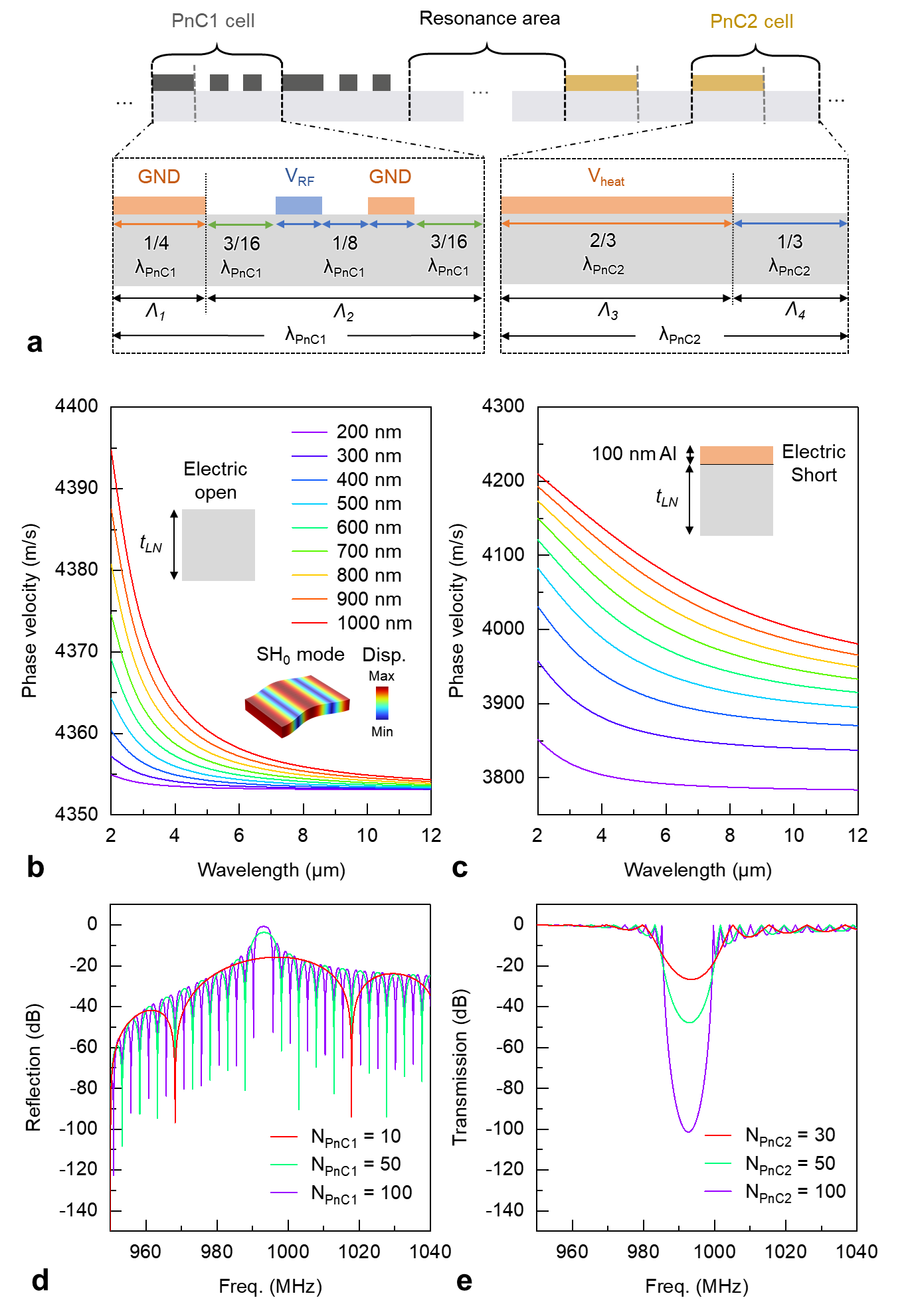


**Figure S1. a)** Detailed structure and design parameters of PnC1 and PnC2. The unit cell length of PnC1 (𝜆*_PnC1_*) is 8 µm, and that of PnC2 (𝜆_PnC2_) is 6 µm. **b)** Simulated phase velocity of the $SH_{0}$ mode in Z-cut LiNbO_3_ under electrically open boundary conditions as a function of film thickness. **c)** Simulated phase velocity of SH_0_ mode in Z-cut LiNbO₃ with a 100 nm Al layer under electrically short boundary conditions, also as a function of LN thickness. **d)** Reflection spectra of PnC1 configurations with n = 10, 50 and 100 unit cells. **e)** Transmission spectra of PnC2 configurations with n = 30, 50, and 100 unit cells.

To elucidate the underlying physical mechanisms of coupling mode formation, we performed three-dimensional finite-element simulations in a quasi-2D approximation (2.5D), assuming uniformity in the lateral (y) direction and setting periodic boundary conditions accordingly. The modeled domain includes SPUDTs, PnC1 reflectors, the central PnC2 region, and suspended air-backed LiNbO_3_. **Figure S2 (a)** illustrates the simulation layout, with low-reflection boundary conditions in the longitudinal direction and periodic conditions in the lateral direction. **Figure S2 (b)** presents the simulated transmission (S_21_) spectra with and without PnC2. In the absence of PnC2, the structure does not support a well-defined bandgap, and the SH wave freely transmits across the device.


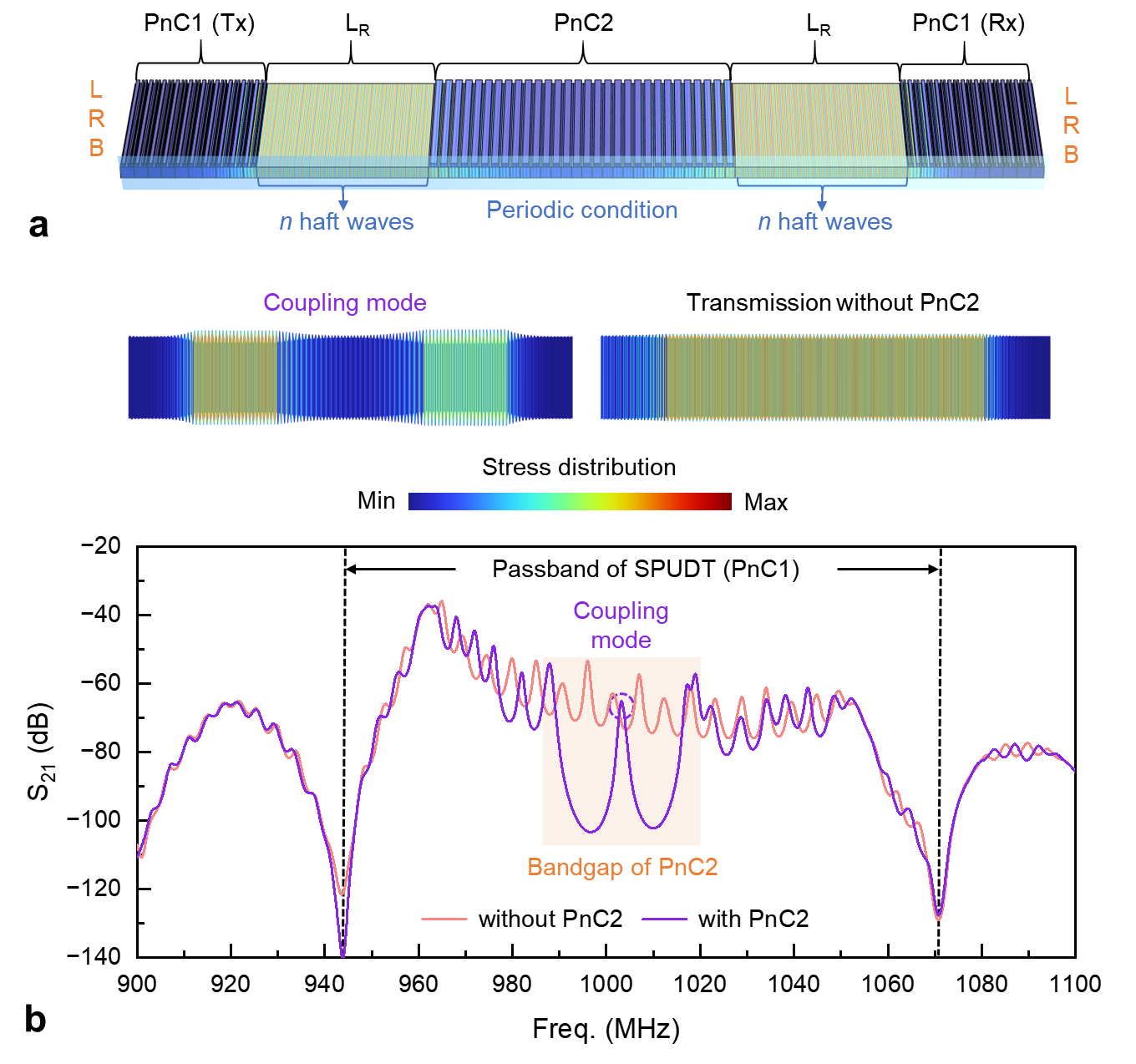


**Figure S2. a)** Simulation setup of the metal–piezoelectric PnC resonator. Low-reflection boundary conditions are applied along the wave propagation (x) direction, while periodic boundary conditions are imposed along the y-direction. For this analysis, wave distribution along the y-axis is neglected. **b)** Simulated transmission (S_21_) spectra comparing devices with 30 PnC2 unit cells and without PnC2. Corresponding stress distributions are shown for the coupling mode (with PnC2) and the transmission mode (without PnC2).

Further simulations were carried out to examine the dependence of resonance behavior on structural parameters. **Figure S3(a)–(b)** shows the S_21_ maps and transmission curves for different resonance cavity lengths L_R_​ (from 97 to 105 μm) at a fixed PnC2 cell number (n = 30). The results confirm Fabry–Perot-type resonance formation, with the number of half-wavelengths directly influencing the number of transmission peaks. **Figure S3 (c)–(d)** explores the effect of varying PnC2 length (number of unit cells from 2 to 60) at fixed L_R_​=101 μm. As cells number of PnC2 decreases, the inter-resonator coupling strength increases, resulting in observable mode splitting and hybridization.


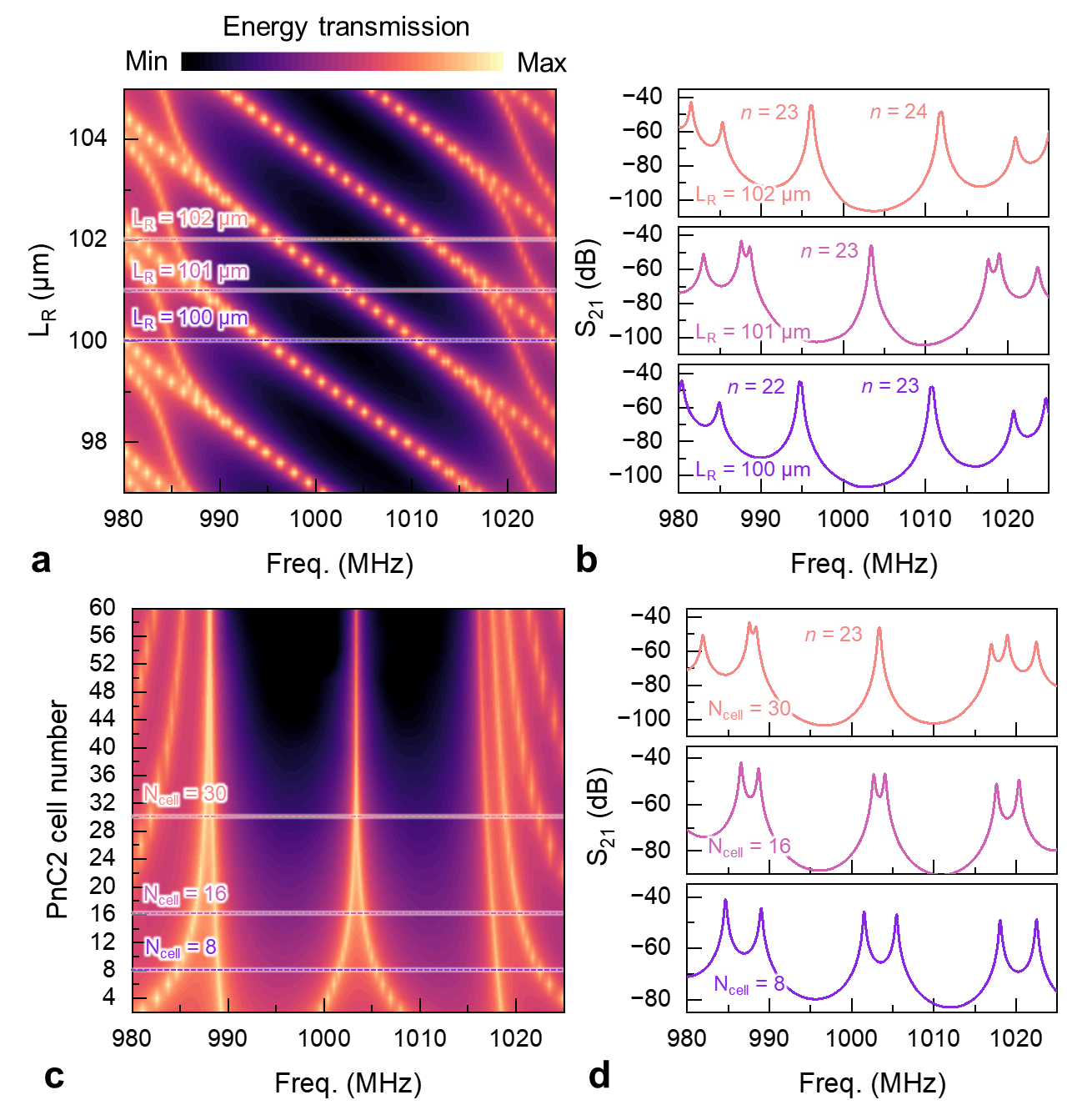


**Figure S3 a)** Simulated transmission (S_21_) map as a function of cavity length L_R_, varied from 97 µm to 105 µm, with the number of PnC2 unit cells fixed at 30. **b)** Transmission spectra for LR = 100, 101, and 102 µm, showing the evolution of resonance peaks and corresponding half-wavelength mode numbers. **c)** Simulated S21 map as a function of the number of PnC2 unit cells (N_cell_) varied from 2 to 60, with L_R_ fixed at 101 µm. **d)** Transmission spectra for N_cell_ =8, 16, and 30, illustrating progressive mode splitting as the coupling strength increases.

**Supplementary Note 3: Effect of LiNbO₃ Thickness Variations on FEM Simulations**

To investigate the impact of LiNbO_3_ thickness variations, we performed finite element method (FEM) simulations by sweeping the film thickness from 250 nm to 350 nm. The simulated transmission coefficient (S_21_) and the extracted quality factor of the trapping mode (quasi-BIC) are shown in **Figure S4 (a)-(b)**. The results indicate that the quality factor exhibits a strong dependence on the LiNbO_3_ thickness, with a pronounced peak in the range of 290–300 nm. This behavior can be understood as follows: thickness variations affect the interference between coupled modes and the confinement ability of the PnC cavity. A change in LiNbO_3_ thickness alters the acoustic velocity contrast with the overlaid Al electrodes and causes frequency shifts, as shown in Supplementary Note 1. These modify the mode dispersion and shift the designed SH-mode frequencies, and causes incomplete interference between ${SH}_{0}^{0}$ and ${SH}_{0}^{2}$mode, thereby weakening the acoustic confinement and reducing the *Q*-factor. Within the 290–300 nm window, the mode alignment is optimal, leading to enhanced confinement and higher simulated *Q*. For numerical convergence of the FEM simulations, a mechanical loss tangent of 1/1000 was applied to both the electrodes and the LiNbO_3_ film. As a result, the simulated Q-factors should be regarded as a relative reference for the effect of thickness variations. In practice, the measured *Q* of fabricated devices exceeds the simulated values, which suggests that the actual mechanical losses of the thinned LiNbO_3_ films are lower than those assumed in the simulations.


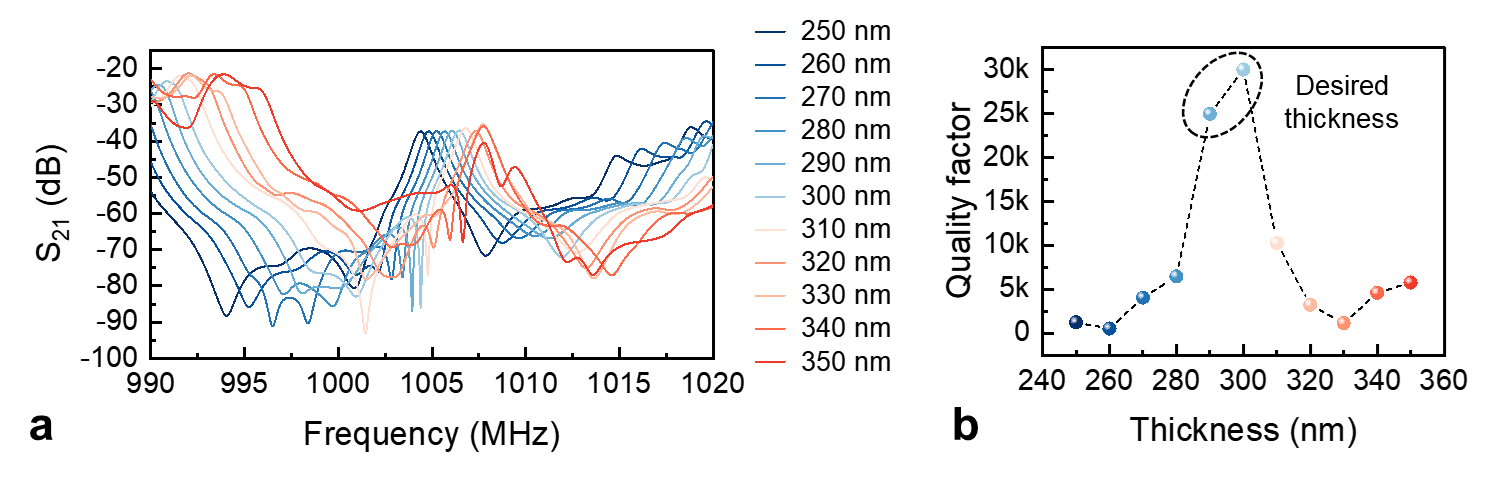


**Figure S4. a) Simulated transmission coefficient S_21_ with LiNbO_3_ thin-film thickness varied from 250 nm to 350 nm. b) Extracted quality factor (Q) of the trapping mode (quasi-BIC) versus film thickness. A peak in Q is observed around 290–300 nm.**

**Supplementary Note 4: Transmission response of the device with PnC2 and control group device**

To validate the influence of PnC2 on acoustic confinement and Q-factor enhancement, we fabricated and measured two sets of devices: one with the PnC2 structure and another control group without it. **Figure S5 (a)–(b)** displays optical images of both devices, showing the presence or absence of the central phononic coupler. Reflection (S_11_) and transmission (S_21_) spectra were recorded using a vector network analyzer. As shown in **Figure S5 (c)–(d)**, the control device without PnC2 exhibits no significant bandgap behavior, and only a single broad resonance is observed. In contrast, the device incorporating PnC2 shows a distinct transmission bandgap near 1 GHz and two resonance peaks within the gap—corresponding to the leaky mode and quasi-BIC mode, respectively.


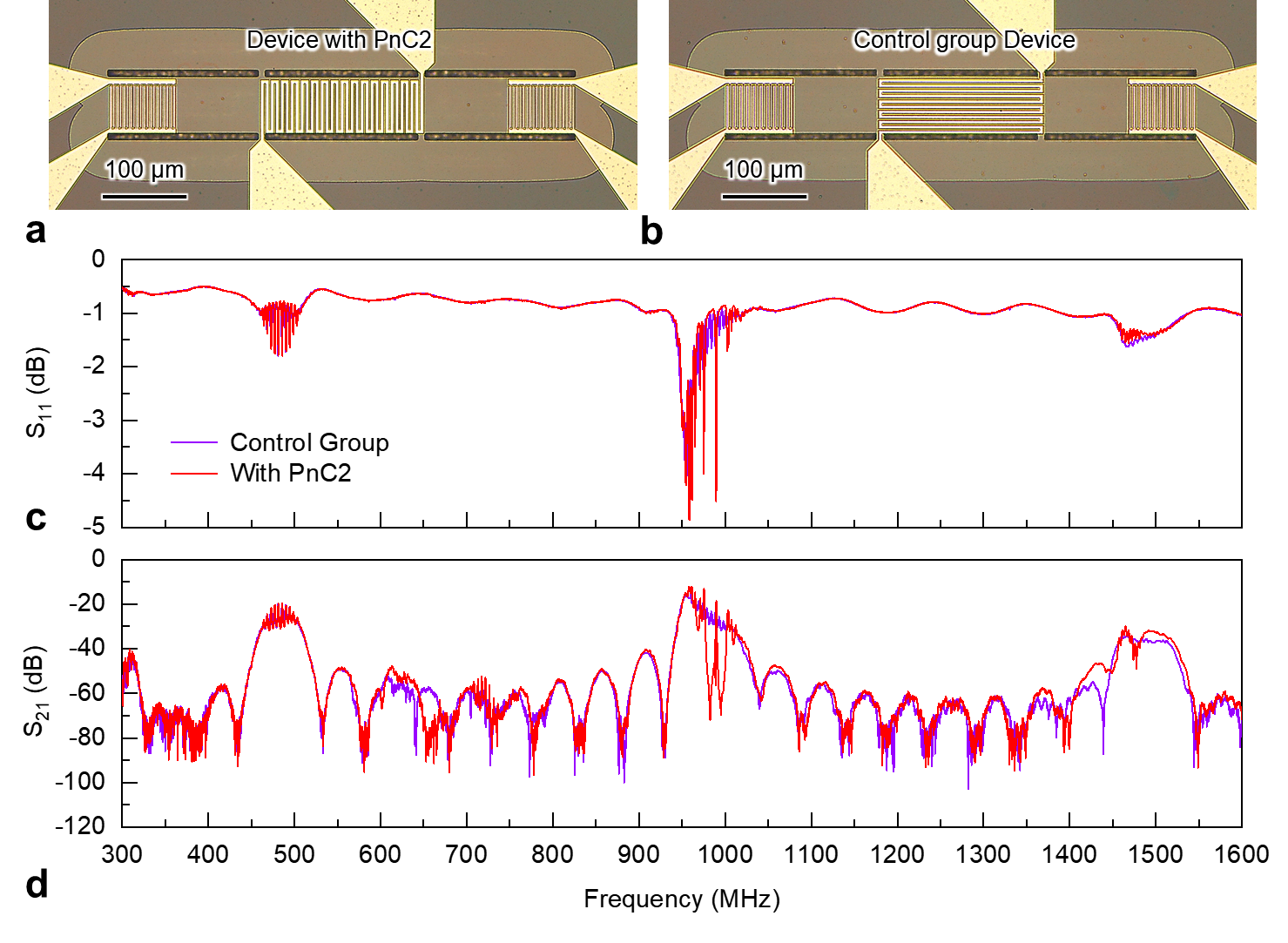


**Figure S5.** **a)** Optical micrograph of the fabricated device with integrated PnC2 structure. **b)** Optical micrograph of the control device without PnC2. **c)** Measured reflection spectra (S_11_​) and **d)** transmission spectra (S_21_​) of both devices. The device with PnC2 exhibits a clear bandgap with a transmission mode centered around 1 GHz, in contrast to the control device which shows no bandgap behavior.

**Supplementary Note 5: Device-to-device reproducibility of *Q*-factor and *f* × *Q* performance**

To evaluate reproducibility, we fabricated and measured five nominally identical devices from the same batch. The extracted quality factors (*Q*) and *f* × *Q* products of both the transmission and quasi-BIC (trapping) modes are summarized in Figure S5. As shown in **Figure S6 (a),** for the transmission mode, we obtained a maximum *Q* of 2091, a minimum *Q* of 2069, with an average *Q* of 2075 and a standard deviation of 9.43. The small variation indicates that the transmission resonance is insensitive to fabrication non-uniformities. In contrast, for the quasi-BIC trapping mode, the *Q* shows larger variation, with a maximum of 64,620, a minimum of 35,803, an average of 49,691, and a standard deviation of 12,196. This pronounced variation is attributed to thickness non-uniformities introduced during the thinning process. Controlling the LiNbO_3_ thickness is critical, as variations increase the acoustic velocity contrast with the Al electrodes, alter mode dispersion, which can cause incomplete mode interference, thereby reducing acoustic confinement and lowering *Q*-factors (see Supplementary Note 3). In our devices, thickness control was successfully achieved, as reflected by the reproducible high-*Q* performance across multiple devices. The *f* × *Q* products for the five devices are shown in **Figure S5 (b)**, with a maximum value of 6.39 × 10^13^ Hz and a minimum value of 3.54 × 10^13^ Hz.


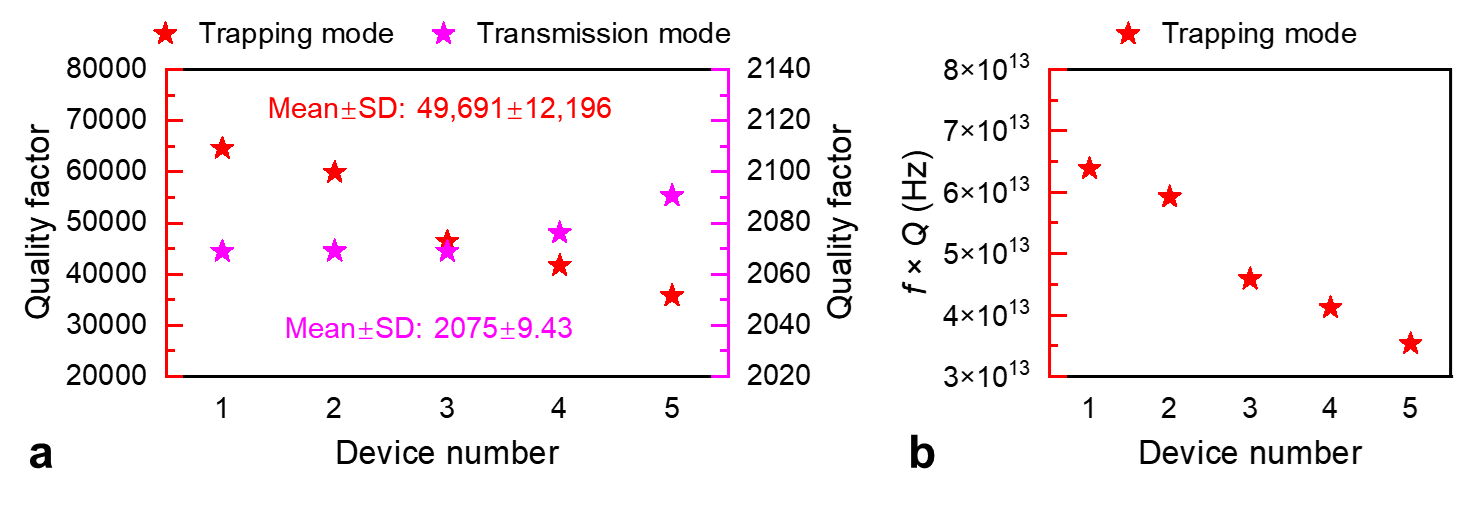


**Figure S6.** **a)** Extracted Q-factors of the transmission mode and quasi-BIC (trapping) mode for devices 1–5. **b)** *f* × *Q* products of the quasi-BIC modes for devices 1–5.

**Supplementary Note 6: AC thermal modualtion of the piezoelectric-metal phononic crystal device**

To characterize the AC thermal modulation of the piezoelectric–metal phononic crystal device, a square-wave signal with an amplitude of 0.5 V and a duty cycle of 50% was applied to the microheater using a waveform generator, while a vector network analyzer (VNA) monitored the corresponding changes in S_21_. The device operated at a frequency of 988 MHz, and modulation frequencies of 50 Hz, 100 Hz, 500 Hz, and 1000 Hz were tested, as shown in **Figure S7(a)**. **Figure S7(b)** presents the transient response under 50 Hz modulation. The rise time, defined as the interval for the S_21_ change to increase from 0% to 90%, was measured to be 1.45 ms, while the fall time, corresponding to the decrease from 100% to 10%, was 1.05 ms. These response times are considerably faster than previously reported values for SAW-based platforms. This improvement is attributed to the suspended LN thin-film structure, which provides excellent thermal isolation and low thermal capacity, thereby enhancing thermal energy efficiency.


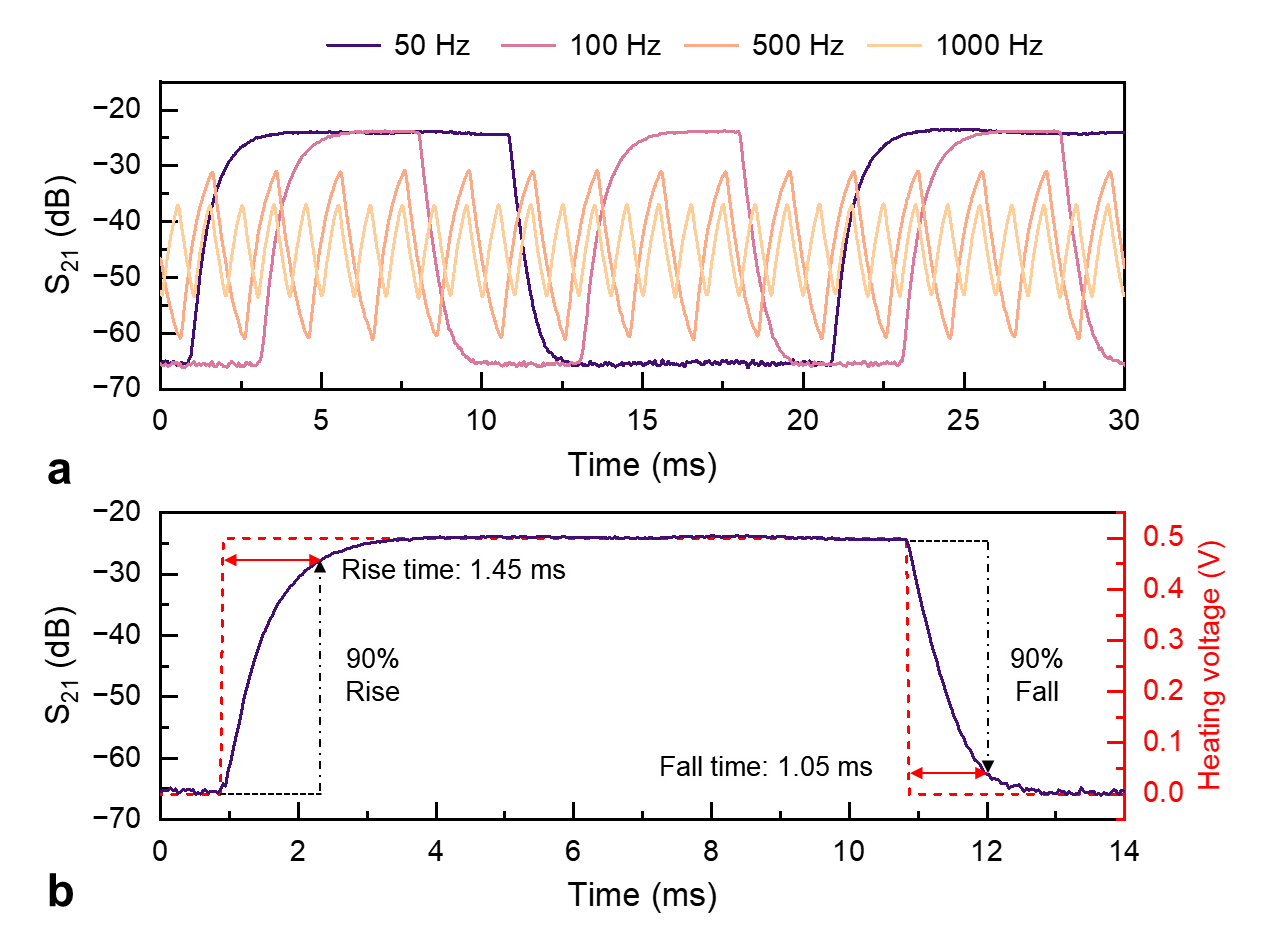


**Figure S7.** **a)** AC thermal modualtion of the piezoelectric-metal phononic crystal device with freqeuncy of 50 Hz, 100 Hz, 500 Hz and 1000 Hz **b)** Transient response of the output phase when a 0.5V square wave is inputted to heater.

**Supplementary Note 7: Comparison with state-of-the-art high-*Q* resonators reported in the literature**

Table S1 Comparison of the state-of-the-art high-*Q* resonator designs

| Refs | Platfrom | Deisgn | Principle | Freq. (GHz) | *Q* | *f* × *Q* (Hz) | Assigned logo |
| --- | --- | --- | --- | --- | --- | --- | --- |
| [55] | Bulk LiNbO_3_ | Shallow etched PnC | Cavity mode | 0.51 | 18,100 | 0.93 × 10^13^ | ● |
|  |  |  |  | 1.02 | 16,700 | 1.70 × 10^13^ | ● |
|  |  |  |  | 3.07 | 6,240 | 1.92 × 10^13^ | ● |
|  |  |  |  | 5.11 | 2,480 | 1.27 × 10^13^ | ● |
| [57] | Bulk LiNbO_3_ | Shallow etched PnC | Topological edge mode | 1.05 | 11,572 | 1.21 × 10^13^ | ● |
| [64] | LiTaO_3_ on SiC | SAW resonator | SH-SAW | 1.16 | 11,000 | 1.27 × 10^13^ | ■ |
| [63] | LiNbO_3_ on SOI | SAW resonator | SH-SAW | 0.95 | 1,316 | 1.25 × 10^12^ | ■ |
| [65] | LiNbO_3_ on sapp. | SAW resonator | SH-SAW | 1.12 | 1,100 | 1.23 × 10^12^ | ■ |
| [66] | LiNbO_3_ on SiC | SAW resonator | SH-SAW | 2.28 | 1,228 | 2.80 × 10^12^ | ■ |
| [63] | LiNbO_3_ thin film | Lamb wave resonator | S_0_ mode | 0.05 | 5,300 | 2.65 × 10^11^ | ▼ |
| [68] | LiNbO_3_ thin film | Lamb wave resonator | A_1_ mode | 1.65 | 3,112 | 5.13 × 10^12^ | ▼ |
| [69] | LiNbO_3_ thin film | Lamb wave resonator | A_1_ mode | 3.25 | 374 | 1.21 × 10^12^ | ▼ |
| [70] | LiNbO_3_ thin film | Lamb wave resonator | A_1_ mode | 5.00 | 340 | 1.70 × 10^12^ | ▼ |
| [54] | AlN on SOI | Shallow etched PnC | BIC mdoe | 2.30 | 10,100 | 2.32 × 10^13^ |  |
| [58] | Bulk GaAs | Deeply etched PnC | BIC mdoe | 2.04 | 2,400 | 4.90 × 10^12^ |  |
| **This work** | **LiNbO_3_ thin film** | **Piezo-Metal PnC** | **BIC mdoe** | **0.989** | **64,620** | **6.39 × 10^13^** | ★ |
